# Supplementary figures and images for: Threat at One End of the Plant: What Travels to Inform the Other Parts?
Source: Int J Mol Sci. 2021 Mar 19;22(6):3152. doi: 10.3390/ijms22063152 (PMC8003533; doi:10.3390/ijms22063152)

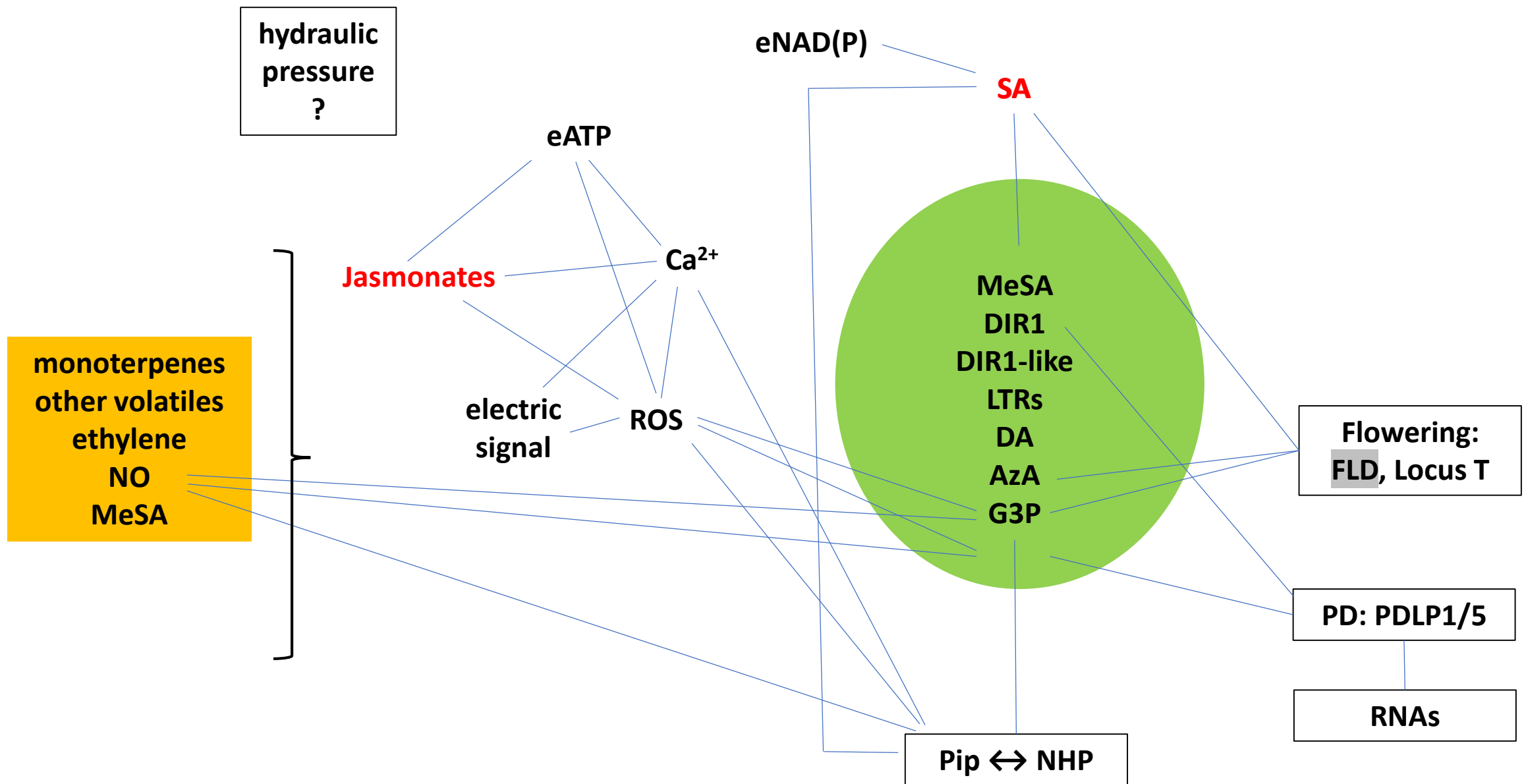

Suppl. Fig. 1

Supplement: Supplementary file 1 [file ijms-22-03152-s001.pdf]
